# Clinical Features of Idiopathic Parotid Pain Triggered by the First Bite in Japanese Patients with Type 2 Diabetes: A Case Study of Nine Patients

**DOI:** 10.1155/2018/7861451

**Published:** 2018-03-29

**Authors:** Masatoshi Chiba, Hiroaki Hirotani, Tetsu Takahashi

**Affiliations:** ^1^Division of Oral and Maxillofacial Surgery, Department of Oral Medicine and Surgery, Tohoku University Graduate School of Dentistry, 4-1 Seiryo-machi, Aoba-ku, Sendai, Miyagi 980-8575, Japan; ^2^Dentistry and Oral Surgery, Osaki Citizen Hospital, 3-8-1 Honami, Furukawa, Osaki, Miyagi 989-6183, Japan

## Abstract

**Objective:**

First bite syndrome, characterized by pain in the parotid region after the first bite of each meal, predominantly develops in patients who have had head and neck surgery. Idiopathic parotid pain (IPP) that mimics first bite syndrome may present in patients without a history of surgery or evidence of an underlying tumor, but its clinical features are unclear. This study characterized the clinical characteristics of IPP in patients with diabetes.

**Study Design:**

A retrospective case review involving the clinical findings and pain characteristics of nine patients with IPP and diabetes who presented to our department between 2013 and 2016.

**Results:**

All the patients were men diagnosed with type 2 diabetes (median age, 43 years). IPP developed unilaterally in seven patients and bilaterally in two. The median intensity of the first bite pain was 8 on a numerical rating scale of 0–10. The trigger factor was gustatory stimuli, and the trigger area was the posterior section of the tongue. Postprandial pain occurred within 1–10 min after meals in six patients.

**Conclusions:**

IPP may be considered a separate disorder, in which the pain characteristics are similar to those of first bite syndrome but the clinical features and pathophysiology are different.

## 1. Introduction

First bite syndrome is a rare pain syndrome that is characterized by severe paroxysmal pain in the parotid region, which is precipitated by the first bite of each meal and diminishes in severity with subsequent mastication [1–7]. First bite syndrome often affects a patient's quality of life and ability to eat. Although the definitive pathogenic mechanism of first bite syndrome remains to be clarified, the most classical hypothesis implicates damage to sympathetic innervation to the parotid gland, leading to denervation hypersensitivity of the myoepithelial cell sympathetic receptors responsible for the parotid pain at the first bite [1–7]. Treatments include medication and surgical intervention; however, their effectiveness has not been well documented [4–6, 8].

First bite syndrome has previously been described as a complication of surgery of the upper cervical region involving the infratemporal fossa, parapharyngeal space, and parotid gland [1–7]. Additionally, four cases of first bite syndrome secondary to a malignant tumor arising from the parotid gland, the submandibular gland, or parapharyngeal space have been documented to date [9–12]. In the recent literature, a small number of single case reports of idiopathic first bite syndrome in the absence of a history of head and neck surgery and without evidence of an underlying malignancy have been reported [13–15]. Except for the characteristic pain pattern, the clinical picture and pathogenesis of idiopathic first bite syndrome are largely unknown. In addition, very few clinical studies have been conducted to quantitatively and comprehensively evaluate first bite syndrome related pain even in postoperative first bite syndrome [6]. Thus, it remains unclear whether idiopathic first bite syndrome has the same diagnostic features as first bite syndrome. For this reason, in the present study, we used the term idiopathic parotid pain (IPP) instead of idiopathic first bite syndrome.

Cases of IPP have been mainly reported in patients with diabetes [13, 14]. Diabetic neuropathy is the most common form of neuropathy in developed countries and may affect almost one-half of the patients with diabetes. Moreover, diabetic autonomic neuropathy can affect any organ innervated by the autonomic nerves. Therefore, in this study, we focused on patients with IPP complicated by diabetes and performed a retrospective case review to investigate the clinical and pain characteristics of IPP.

## 2. Patients and Methods

We conducted a retrospective case study to determine the clinical findings and pain characteristics of IPP. The study was performed in accordance with the standards of the Declaration of Helsinki and was approved by our institutional review board (2016-3-25).

### 2.1. Patients

Fourteen patients with IPP who presented to the authors' orofacial pain clinic between January 2013 and December 2016 were enrolled in this study. All medical records were reviewed retrospectively. Patients received a definitive diagnosis of IPP if they met the following criteria: (1) parotid pain that coincided with the first bite of every meal and subsided over the next several bites; (2) the absence of salivary gland, masticatory muscle, temporomandibular joint, or dental pathology capable of causing the facial pain; (3) no history of injury or surgery in the head and neck region; and (4) no evidence of tumor arising from the salivary glands or the parapharyngeal space. Five patients were excluded as they did not have diabetes, and therefore, nine patients were finally included in this study.

### 2.2. Data Collection

We analyzed patient characteristics (age, sex, and medical history), clinical symptoms, laboratory findings (glycosylated hemoglobin [HbA1c], serum amylase, and coefficient of variation of R-R interval [CV_R-R_]), imaging findings, pain characteristics, and course of IPP.

The parotid glands, masticatory muscles, and temporomandibular joints were evaluated by a physical examination. Sensory abnormalities in the trigeminal nerve were documented based on the patient's response to light touch. Parotid pain elicited by the application of a sour solution to the tongue was recorded. Whether parotid pain was triggered by biting a cotton swab was also recorded.

Information about the status of diabetes before the onset of IPP was obtained from the patients' physicians. Diabetic control was considered good when HbA1c was less than 7 [16]. Cardiovascular autonomic dysfunction was suggested if CV_R-R_ was less than 2%. All patients underwent panoramic radiography and magnetic resonance imaging (MRI) of the head and neck on a 1.5-T imaging system. MRI studies were reevaluated with a particular focus on the salivary glands, parapharyngeal space, temporomandibular joints, and course of the trigeminal and glossopharyngeal nerves.

Patients were asked to rate the severity of pain intensity and the degree to which pain interfered with their eating during the past one week using a 0–10 numerical rating scale (NRS), with “0” denoting “no pain” and “10” denoting “pain as bad as you can imagine” with respect to pain intensity and with “0” denoting “does not interfere” and “10” denoting “completely interferes” with respect to interference with eating. The improvement in IPP was based on the reduction in the degree of parotid pain.

### 2.3. Statistical Analysis

Continuous variables were expressed as median and range. Wilcoxon signed rank test was conducted to evaluate the changes in pain level before and after treatment, and a* P* value < 0.05 was considered to be significant. All statistical analyses were performed using the JMP statistics software program version 12 for Macintosh (SAS Institute Inc., Cary, NC, USA).

## 3. Results

### 3.1. Patient Characteristics of IPP

Patient characteristics are shown in Table 1. All the patients were Japanese men with a median age of 43 years (range, 38–66 years). IPP onset was sudden with no prodromal symptoms, and the median duration of the symptoms was 2 months (range, 1–4 months). IPP developed unilaterally in seven patients and bilaterally in two patients, but did not occur simultaneously. A total of 11 parotid glands in nine patients were examined.

All patients had type 2 diabetes. The median duration of diabetes was 7 years (range, 0.5–20 years). Two patients received no diabetes care (cases 2 and 9). Seven patients were treated with several types of antidiabetic medications, none of which had a side effect of parotid pain; however, diabetic control was poor before the onset of IPP. HbA1c of three patients (cases 3, 6, and 8) was less than 7 at the first medical examination, but was more than 8 during the several months before the onset of IPP. In addition to the treatment of diabetes, severe IPP made eating difficult, consequently leading to a 6% significant decrease in HbA1c within two months in one patient (case 3). One patient was diagnosed with diabetic polyneuropathy (case 4), one patient had diabetic cataract (case 3), one patient had a history of foot cellulitis (case 6), one patient had renal disease (case 8), and one patient had diabetic retinopathy (case 9). Three patients had hypertension that was treated with blood pressure-lowering drugs (cases 4, 5, and 8). All patients were misdiagnosed as having parotitis, temporomandibular disorders, or dental pain by their dentist or physician.

### 3.2. Clinical Characteristics of IPP

The clinical findings of this study are summarized in Table 1. Physical examination revealed that the parotid regions were not visibly swollen, and salivary flows were unrestricted in all patients. The median maximal incisal opening was 52 mm (range, 40–60 mm). In all the patients, routine neurological examination revealed no objective sensory deficit or paresthesia of the orofacial region. Furthermore, when applying mild pressure over the parotid region, only the affected parotid glands were tender.

The median of HbA1c and serum amylase concentration was 8% (range, 5.4–10.4%) and 55 U/l (range, 37–73 U/l; normal range, 37–125 U/l), respectively. CV_R-R_ was less than 2% in two of the six patients who underwent the test. Radiological examinations, including MRI, revealed no evidence of any underlying lesion of the head and neck in the any of the patients. Fat-suppressed T2-weighted images showed no increase in signal intensity in the parotid glands and no dilatation of ducts in any of the patients. Three patients underwent repeat MRI examinations of the head and neck, which confirmed the absence of abnormalities.

### 3.3. Pain Characteristics of IPP

Patients' pain characteristics are summarized in Tables 2 and 3. The first bite pain of the patients was consistent with the pain pattern reported by patients with postoperative first bite syndrome. Parotid pain occurred immediately at the first introduction of various foods into the mouth even in the absence of mastication, but the pain evoked by chewing food was stronger than the pain precipitated by introducing food into the mouth. Acidic food was most effective in eliciting the pain. In addition, the pain was stronger with strong-tasting foods than with mild-tasting foods. The pain invariably occurred with the first bite of each meal and subsided within a minute of continued mastication. Once the pain had passed, the patients could continue chewing without further pain. However, the pain returned with the first bite of the next meal.

First bite pain was confined to the parotid region in seven patients and radiated to the preauricular region in two patients. In all patients except for one patient (case 9), the parotid pain was evoked by applying a sour solution with an applicator to the posterior section of the ipsilateral tongue but not to the contralateral tongue. However, jaw movements and biting a cotton swab few times did not evoke the pain. Based on the NRS, the median intensity of the first bite pain was 8 (range, 5–10). The median degree of interference with eating due to pain was 9 (range, 3–10). The quality of the first bite pain was described as stabbing (three patients), sharp (two patients), smarting (two patients), or tightening (two patients).

In six patients, the postprandial pain occurred on the ipsilateral side of location of the first bite pain within 1 to 10 min after meals. This pain was confined to the parotid region in one patient and the preauricular region in one patient. In the other four patients, the parotid pain radiated to the preauricular, temporal, or occipital regions. The median postprandial pain intensity was 8.5 (range, 6–10) on the NRS. The postprandial pain lasted from 2 to 30 min. The quality of the postprandial pain was described as stabbing (two patients), smarting (one patient), burning (one patient), or pricking (one patient); data regarding quality of postprandial pain were missing from the sixth patient's chart.

### 3.4. Clinical Course of IPP

One patient could not receive therapy owing to sudden change of residence (case 5). The remaining eight patients underwent dietary modification involving ingestion of bland food, avoidance of acidic foods, and chewing on the unaffected side. One patient responded to this dietary modification (case 9). The other seven patients required pharmacologic treatment mainly consisting of pregabalin or neurotropin (a drug used to treat postherpetic neuralgia in Japan). The two patients with untreated diabetes were referred to a diabetes specialist and underwent treatment for diabetes.

The first bite pain improved in all eight patients, who underwent treatment, and two of these patients reported complete relief from symptoms. The median pain intensity of the first bite pain before and after treatment improved from 8 (range, 5–10) on the NRS to 2.3 (range, 0–5) at the final follow-up visit, which was a significant difference (*P* < 0.01) (Table 2). The postprandial pain completely disappeared in all six patients, and an improvement in postprandial pain always preceded an improvement in the first bite pain. The median pain intensity of the postprandial pain improved significantly from 8.5 (range, 6–10) on the NRS to 0 at the final follow-up visit (*P* = 0.03). All the patients were generally able to eat a normal diet and reported the disappearance or decline of parotid tenderness.

## 4. Discussion

In this study, we aimed to clarify the clinical features of IPP in patients with diabetes. We demonstrated that first bite pain in the parotid gland was triggered by nonnoxious gustatory stimuli and aggravated by mastication and that the trigger area was located on the posterior section of the ipsilateral tongue. IPP was severe enough to impair a patient's ability to eat. Of particular note is that all the affected parotid glands were sensitive to slight pressure, IPP developed bilaterally in two patients, and postprandial pain occurred in six patients.

The results of the current study showed that the first bite pain of IPP was severe (a median score of 8 on the NRS) and lasted for a short duration (less than 1 min). The first bite pain of IPP was triggered by gustatory stimuli; acidic sialogogic foods triggered particularly strong pain. A recent retrospective study that evaluated 17 patients, who developed postoperative first bite syndrome after upper cervical surgery, reported that the pain severity ranged from 6 to 10 on the NRS, the duration of pain ranged between 3 seconds and throughout the meal, and the factor triggering pain was chewing and/or simple contact with food; these foods were always acidic [6]. These pain characteristics are similar to those described in our study in many respects. Our study demonstrated that the trigger area was the posterior one-third of the ipsilateral tongue; however, the trigger area in postoperative first bite syndrome has not yet been determined. These findings indicate that, in IPP, the taste sensation is transmitted via the glossopharyngeal nerve and evokes salivary secretion through the taste-salivary reflex [17], leading to the first bite pain in the affected parotid gland.

There are three main differences between the symptoms of IPP and those of postoperative first bite syndrome. First, in our patient group, all affected parotid glands were sensitive to slight pressure. All other clinical findings were normal, parotid imaging was unremarkable, and serum amylase was not elevated. We therefore ruled out the possibility of the inflammation or anatomical obstruction of the affected parotid glands. These findings indicate that the affected parotid glands were hypersensitive to mechanical stimuli, suggesting peripheral sensitization in or around the parotid glands. Interestingly, the improvement in the first bite pain of IPP was likely associated with the absence or decline of parotid tenderness. Second, our findings showed that the postprandial pain of IPP was of the same intensity as that of first bite pain, with a longer duration of pain, and larger affected area of pain than the first bite pain. This postprandial pain has not yet been described in postoperative first bite syndrome or malignant tumor-associated first bite syndrome [1–7, 9–12]. The present study could not elucidate the pathogenesis that underlies the onset of the postprandial pain. However, the postprandial pain of IPP responded well to pregabalin or neurotropin, which are effective for neuropathic pain. In addition, the postprandial pain of IPP was largely located in the distribution of the auriculotemporal nerve, which innervates the parotid glands. These findings indicate the possibility that neuropathic pain of the auriculotemporal nerve was responsible for the postprandial pain of IPP. Third, unlike postoperative first bite syndrome, which always develops in the ipsilateral surgical side [1–7], IPP in our patients could develop bilaterally, suggesting that the cause of IPP may not be local, but systemic. In part, these differences in symptoms could be explained by the difference in etiologies of IPP and first bite syndrome.

The exact pathogenesis of first bite syndrome remains to be clarified, because the absence of animal studies and the rarity of first bite syndrome hinder a thorough understanding. According to Netterville et al. [1], who first postulated the pathogenesis of first bite syndrome, this syndrome is secondary to the loss of sympathetic innervation to the parotid gland. This would result in denervation hypersensitivity of the sympathetic receptors that control the myoepithelial cells in the parotid gland. With oral intake, the secreted parasympathetic neurotransmitter cross-stimulates the sympathetic receptors, causing supramaximal contraction of the myoepithelial cells and first bite pain in the parotid region. This hypothesis, also supported by many investigators, can explain all etiologies of first bite syndrome [2–11]. However, this etiological mechanism of first bite syndrome cannot explain the pathogenesis of IPP, because there is a little evidence that the sympathetic innervation of the parotid gland of IPP was affected in our patients. Furthermore, while the gustatory stimuli-evoked facial pain has been reported previously [18–21], the IPP in our case series differs from the gustatory stimuli-evoked pain described in previous reports. Thus, the pathogenesis of IPP in our study remains to be elucidated.

The cause of the IPP cases in our study remains unknown. With regard to comorbidities that may contribute to IPP, nine out of 14 IPP patients were complicated with type 2 diabetes and presented hyperglycemia before the onset of IPP. Hyperglycemia is well recognized as a major cause of diabetic neuropathy [22]. Small fibers (myelinated A*δ* and unmyelinated C fibers, including the autonomic nerves) are preferentially affected in the early stages of diabetes [23]; however, small fiber neuropathy is overlooked in many patients. Physicians were aware of painful diabetic neuropathy in only 36.4% of patients with the condition in Japanese clinical practice [24]. In this study, only one patient was diagnosed as having diabetic polyneuropathy by his physician, and two patients exhibited cardiovascular autonomic neuropathy. Diabetes is a common disease, whereas IPP is rare. Therefore, while we believe that there might be an association between diabetic neuropathy and the pathogenesis of IPP in our patients, it is unlikely that diabetic neuropathy alone contributed to the development of IPP. Further studies are required to elucidate the association between diabetic neuropathy and IPP.

In our study, all the patients with IPP and diabetes were men, suggesting that men are more likely to develop IPP. This may be attributed to the fact that, compared to women, men have more severe symptoms of diabetic neuropathy, have a higher incidence of diabetic neuropathy, and develop diabetic neuropathy earlier than women [25, 26]. On the other hand, a retrospective study of 499 patients undergoing surgery of the infratemporal fossa, parotid gland, and/or parapharyngeal space reported that the female gender was significantly associated with the development of first bite syndrome [4].

The recurrent and severe pain associated with IPP sometimes prevents patients from eating, leading to inadequate nutritional intake and interference with diabetes management. In our opinion, impairment in eating and systemic health necessitates immediate treatment of IPP. Although limited data exist regarding the efficacy of pharmacologic treatment of postoperative first bite syndrome, anticonvulsants such as carbamazepine, pregabalin, and gabapentin used alone or in combination with tricyclic antidepressants have been shown to decrease the severity and/or duration of the first bite pain [5, 8]. In our study, in the seven IPP patients who were treated with medication, administration of either pregabalin or neurotropin reduced the severity of first bite pain and completely relieved postprandial pain.

This study has a few limitations. First, it was difficult to compare the clinical features of IPP between patients with and without diabetes because of the small number of cases. Second, the relationship between IPP and diabetes was speculated. Third, it was difficult to evaluate the therapeutic effects of the treatment for IPP, because patients received multiple treatments. Therefore, going forward, further studies involving a large number of patients are required to further elucidate the relationship between type 2 diabetes, gender, and IPP.

## 5. Conclusions

The present study showed that IPP in Japanese patients with type 2 diabetes presents as first bite pain consistent with first bite syndrome, that IPP was induced by gustatory stimuli and aggravated by mastication, and that the trigger area was the posterior section of the ipsilateral tongue. The present study also demonstrated that the affected parotid glands were sensitive to slight pressure and that IPP developed bilaterally in two patients and postprandial pain occurred in six patients. IPP in patients with type 2 diabetes may be considered a separate disorder, in which the pain characteristics are similar to those of first bite syndrome but the clinical features and pathophysiology are different. Although IPP is a rare disease, pain clinicians should consider IPP as a differential diagnosis in patients presenting with first bite pain in the parotid region.

## Figures and Tables

**Table 1:** Clinical characteristics of the patients with idiopathic parotid pain.

Case	Age (years)	Sex	Affected side	Duration of idiopathic parotid pain (months)	Duration of diabetes (years)	HbA1c (%)	Serum amylase (U/l)	CV_R-R_ (%)
1	45	Male	Left	1	3	7.4	50	2.49
2	38	Male	Bilateral	4	10	10.4	73	6.02
3	41	Male	Bilateral	2.5	5	6.7	37	1.42
4	39	Male	Right	3	9	9.8	56	3.24
5	50	Male	Right	1	10	9.8	39	Untested
6	38	Male	Right	1	0.5	6.0	44	Untested
7	66	Male	Left	2	20	8.0	71	Untested
8	57	Male	Right	2.5	7	5.4	55	3.82
9	43	Male	Left	1	2	8.5	62	1.55

HbA1c, glycosylated hemoglobin; CV_R-R_, coefficient of variation of R-R interval.

**Table 2:** Characteristics of first bite pain among patients with idiopathic parotid pain.

Case	Pain site	Pain intensity (NRS: 0–10)	Pain quality	Pain duration	Degree of interference with eating (NRS: 0–10)	Posttreatment pain intensity (NRS: 0–10)
1	Parotid region	9	Stabbing	Less than 40 seconds	9	0
2	Parotid and preauricular region	8	Smarting	20 seconds	6	4
3	Parotid and preauricular region	6	Smarting	Less than 30 seconds	10	1.5
4	Parotid region	6	Stabbing	A few seconds	5	3
5	Parotid region	7	Tightening	Less than 1 minute	9	No treatment
6	Parotid region	8	Sharp	Less than 1 minute	10	0
7	Parotid region	10	Sharp	A few seconds	8	4
8	Parotid region	10	Stabbing	A few seconds	10	5
9	Parotid region	5	Tightening	A few seconds	3	0

NRS, numerical rating scale.

**Table 3:** Characteristics of postprandial pain among patients with idiopathic parotid pain.

Case	Pain site	Pain intensity (NRS: 0–10)	Pain quality	Pain duration (minutes)	Onset of pain after meal (minutes)
1	Preauricular region	9	Stabbing	2	5–10
2	Parotid, preauricular, and occipital region (right side)	6	Smarting	5	1-2
3	Parotid, preauricular, and occipital region	10	Burning	10	2-3
4	Absent				
5	Parotid, preauricular, and temporal region	10	Pricking	10	5–10
6	Parotid region	7	*∗*	10	5–10
7	Absent				
8	Parotid and temporal region	8	Stabbing	30	1-2
9	Absent				

^*∗*^Missing data; NRS, numerical rating scale.

## Data Availability

Our clinical study was a case study of nine patients, and the all data supporting the results were shown in Results including the tables.
